# A qualitative exploration of the psychological needs of women survivors of rape in Iran

**DOI:** 10.1186/s40359-023-01332-x

**Published:** 2023-10-02

**Authors:** Leila Asadi, Mahnaz Noroozi, Hajar Salimi, Fardin Mardani, Sara Jambarsang

**Affiliations:** 1 Student Research Committee, School of Nursing and Midwifery, Isfahan University of Medical Sciences, Isfahan, Iran; 2 Research Center for Nursing and Midwifery Care, Shahid Sadoughi University of Medical Sciences, Yazd, Iran; 3 Department of Midwifery and Reproductive Health, School of Nursing and Midwifery, Isfahan University of Medical Sciences, Isfahan, Iran; 4 Behavioral Sciences Research Center, Department of Psychiatry, Isfahan University of Medical Sciences, Isfahan, Iran; 5 Forensic Medicine Research Center, Isfahan, Iran; 6 Research Center of Prevention and Epidemiology of Non-Communicable Disease, Department of Biostatistics and Epidemiology, School of Public Health, Shahid Sadoughi University of Medical Sciences, Yazd, Iran

**Keywords:** Psychological needs, Rape, Survivor, Woman, Qualitative study

## Abstract

**Background:**

Rape is one of the principal forms of sexual violence leading to numerous psychological consequences for women. Providing effective psychological services to women survivors of rape requires identifying and considering their real needs. This study aimed to explore the psychological needs of women survivors of rape.

**Methods:**

In this exploratory-descriptive qualitative study, the participants consisted of 19 women survivors of rape and 20 people with experience in providing services to survivors of rape, selected via purposive sampling method in Isfahan, Iran. In-depth individual semi-structured interviews and field notes were used to collect data, which were then analyzed using the conventional content analysis method.

**Results:**

Based on the analysis of the interviews, the psychological needs of women survivors of rape were classified into three main categories: facing psychological problems, attention to emotional reactions, and the need to accept and adapt to reality.

**Conclusions:**

The results revealed that women survivors of rape have different psychological needs. Thus, to meet these needs, supportive and psychological interventions can be considered at both individual and environmental levels. Also, eliminating gender stereotypes affecting the occurrence of rape in the dimensions of Iranian culture can lead to the liberation of the society from the culture of rape.

## Background

Sexual violence against women in various forms violates reproductive and sexual rights and is recognized as a human rights problem in both national laws and international documents. According to World Health Organization (WHO), 6% of women aged 15 and older have been subjected to rape at least once over their lifetime [1–4]. Rape is a complex problem with many physical and psychological consequences for the survivors. Rape can lead to genitourinary, nervous, cardiovascular, respiratory, digestive, and autoimmune complications [5–7], along with numerous psychological problems such as lifelong anxiety, depression, post-traumatic stress disorder (PTSD), eating and sleep disorders, and suicide attempt [8]. Psychological pressure can also increase the re-victimization risk for rape survivors [9]. Thus, survivors of rape need immediate measures and interventions due to its numerous and irreparable complications [3]. In a study, Widanaralalage et al. showed that male survivors repeatedly face stigma and hostility, and there are barriers to their access to effective medical care and reporting to the police. They described feeling re-traumatized after reporting, with officers’ investigative decisions/actions seen as attacks on their credibility as rape victims [10]. Jewkes et al. believed that rape stigma, both external and self-stigmatization (self‐blame), is associated with adverse health outcomes, and rape survivors may benefit from gender‐empowering psychological support that addresses blame and shame [11]. Thus, it is essential to understand the needs of rape survivors and provide them with the required support since the lack of emotional and social support based on their needs results in their psychological trauma [12].

A deeper psychological look at rape survivors as those suffering from multiple psychological trauma and providing them with effective psychological and support services require identifying their real psychological needs. Further, recognizing rape as a violation of health and human rights necessitates attention and understanding of the needs of the survivors, based on which professional services are provided [13]. According to Widanaralalage et al., service providers’ experiences indicated that the role of masculinity and social stigma permeated participants’ accounts, with negative stereotypes and male rape myths influencing reporting, access to services, and survivors’ coping mechanisms [14]. Also, research has shown that 90% of rape survivors will be deprived of optimal services in the absence of structured care that can meet the needs of this population; thus, investigating and understanding the experiences and needs of survivors of rape is a prerequisite to providing them with high-quality services [15].

The statistics and consequences of various types of sexual violence, including rape, are usually underreported in Iran due to strong cultural concerns and more blame on the women survivors than the perpetrators. Thus, rape survivors, families, and society witness the consequences of rape to a greater extent in Iran. Considering the different aspects of experiencing rape, determining the psychological needs of women survivors of rape could provide an appropriate context for systematic, comprehensive, cultural-based interventions as well as caring programs in society. Since qualitative research is an approach for discovering and describing individual’s experiences and giving meaning and understanding to them [16], the present qualitative study was conducted to explore the psychological needs of women survivors of rape.

## Methods

This exploratory-descriptive qualitative study is part of a mixed methods study that was conducted between November 2021 and August 2022.

### Participants

The participants included 19 women survivors of rape and 20 people with work experience providing services to women survivors of rape (Tables 1 and 2). Purposive sampling was used to select the women survivors of rape, which was then continued with the strategy of maximum variety in terms of age, occupation, education, marital status, number of pregnancies and deliveries, time past the rape, age at the time of the rape, and the relationship with the offender. Purposive sampling was also used to select service providers and was then continued with the strategy of maximum variety in terms of work experience.

**Table 1:** Characteristics of women survivors of rape

Age (years)	21–45
Age at the time of rape (years)	21–44
Time after rape (months)	6–20
Religion	Muslim (19)
Education	High school (1), diploma (7), B.S. (10), M.S. (1)
Occupation	Housewife (6), Employee (3), Service job (2), Unemployed (6), Self-employed (2)
Marital status	Single (13), Married (6)
The relationship between the survivor and the offender	Acquaintance (13), stranger (6)
Number of offenders	One (18), Two (1)

**Table 2:** Characteristics of other participants in the study

Age (years)	26–56
Gender	Female (19), Male (1)
Work experience (years)	2–29
Expertise	Obstetricians (2), Reproductive health specialist (2), Midwife (3), Lawyer (2), Emergency medicine specialist (1), Infectious diseases specialist (1), Psychiatrist (3), Psychologist (2), Social worker (1), Forensic medicine specialist (2), Physician (1)

### Settings and recruitment

In the present study, an effort was made to select the participants through all the centers and places to which rape survivors could refer. This would allow more women survivors of rape to be accessed. In addition, sampling from different settings allowed participants with the maximum variety to enter the study and select women with different characteristics, experiences, and views about the subject of the study. In this way, the richness of the findings would be enhanced, and a better understanding of the psychological needs of women survivors of rape would b.

e provided. Participants were accessed through behavioral diseases counseling centers, private offices of physicians and specialists (gynecology, infectious diseases, and psychiatry), psychology and counseling centers, offices of midwives and reproductive health specialists, centers for counseling and midwifery services, counseling centers for vulnerable women (affiliated with the deputy of health department), drop-in centers (DIC) affiliated with the Welfare Organization, drug rehabilitation centers, social emergency centers, women rehabilitation centers affiliated with the Welfare Organization, social deputy of law enforcement police, crime prevention unit affiliated with the Judiciary, vice-chancellor in student and cultural affairs (offices of dormitories and faculties), emergency and genecology clinics in hospitals, and forensic medical centers in Isfahan, Iran.

The first author (LA) recruited participants through face-to-face visits or phone calls. There was no compulsion for participants to enter the research, and they all participated voluntarily. Note that before making phone calls with the participants, permission to contact them was granted by the officials of the centers. After reaching eligible participants, none refused to participate in the study. The first author (LA) had no role or relationship with the centers or participants.

### Inclusion criteria

The inclusion criteria for the women survivors of rape were informed consent and willingness to participate in the interviews, the ability to communicate and express rape experiences, Iranian nationality, age of ≥ 21 years at the time of conducting research, at least six months and most two years passing the event [17, 18], no known psychological disorders or a history of psychiatric treatment before the rape or at present (according to the participant’s report), no prior experience of rape, and no experience of unfortunate events in life (death of a family member, etc.) over the last three months. Willingness to participate in the interviews was the inclusion criterion for service providers.

### Data collection

Data were collected using semi-structured in-depth interviews and filed notes. The first author (LA) conducted the interviews and filed notes. She had 11 years of working experience in midwifery and was a Ph.D. candidate in reproductive health at Isfahan University of Medical Sciences. Other authors had previous experience in qualitative paper/report writing and interviewing. Writing down initial preconceptions about the target group based on the investigators’ previous working experiences with women survivors of rape led the research team to produce the interview guide. Questions, prompts, and guides were piloted in two pilot interviews. All participants were given an information sheet and signed an informed consent form before the interviews started. The following questions were raised at the beginning of the interviews with the women survivors of rape: “*Could you please explain this incident (rape)? What psychological problems have you faced since this incident? What were some of your perceived psychological needs?* (Table 3). The illustrations and interpretations provided by participants directed the interview process. Interviews with service providers started with the general question: *“What are some psychological needs of women survivors of rape?”* and then were directed by the explanations and interpretations of participants. No one else was present at the interview besides the participants and the researchers. Most of the interviews were conducted in one session. Sessions with participants who provided thorough information about the topic were divided into two sessions to avoid a long and boring interview. All interviews were digitally recorded using an MP4 device. No participant wanted the voice recorder not to be used, and this information was included in the informed consent. In this study, 39 individual interviews were performed. The interviews (45–60 minutes) were conducted in places preferred by the participants, such as parks. Since in qualitative data analysis, meanings are generated through interpretation, in this sense, the decision about the number of samples and stopping data collection was situational and subjective [19].

**Table 3:** Interview schedule with representative questions

Section	Representative questions
Initial rapport building	So (alias), how old are you? What are your current circumstances (e.g., job, education level)?
Free recall about incident (optional*)	Thank you for telling me a bit about yourself. Now I would like to ask whether you can tell me anything you’d like to about this incident (rape). If you’d prefer not to take this approach, don’t worry, I have questions we can start with instead
Psychological problems	What psychological problems have you faced since this incident?
Perceived psychological needs	What were some of your perceived psychological needs since the incident?
Needs for disclosure	After the incident, to whom and how did you want to disclose the matter? What did you need to disclose the incident?
Needs related to family members, spouse, etc.	How could your family member and that of your husband support you? What would you like them to do for you? Was there anyone other than family members and your husband whose support you think you needed? What help could they provide? Please explain?
Needs for present and future life	What did you think about the present and future of your life?
Conclusion	What advice would you give to a woman who experiences this incident?

### Data analysis

Data were analyzed using qualitative content analysis known as the Graneheim and Lundman approach [20]. The data were analyzed manually where no software was used. The first author (LA) regularly and promptly transcribed the interview data. Then, the interviews were examined repeatedly to achieve a thorough understanding and to code the sentences and phrases using the inductive method. Coding was done by the first author (LA), with a subset of 10% of the transcripts coded independently by the second author (MN) using the developed coding frame. A codebook was developed by the first author (LA) based on the research objectives and interview guide, after which it was shared with the research team. Coding discrepancies were resolved through discussion and consensus. In the next step, similar codes were integrated, grouping those with the same meaning and forming sub-categories. The main categories were finally obtained by comparing sub-categories and grouping those with similar or related concepts.

### Rigor and trustworthiness

To ensure the rigor and trustworthiness of the data, the criteria suggested by Lincoln and Guba, including credibility, confirmability, dependability, and transferability, were used [21]. To enhance the credibility of the data, 9 months was spent on data collection. In-depth interviews (at different times and places) and field noting were carried out, and a selection of participants with maximum variety was used. To boost confirmability, transcripts were returned to participants for comments or correction, where the coded interviews were shared with five participating women survivors of rape at different sessions, and their opinions were sought; so that member checking could be obtained. To augment dependability, the opinions of five experts were used to adjust and ensure the consistency of the coded data with what was stated by the participants (external check). In the present study, to increase transferability, the findings of the study were presented to three women survivors of rape with similar characteristics to participants who did not participate in the study to judge the similarity of the data with their own experiences.

### Ethical considerations

Research approval was obtained from the ethics committee of Isfahan University of Medical Sciences (approval code: IR.MUI.NUREMA.REC.1400.133). The reasons for the study were explained before each interview. Informed consent was taken from the participants, who were assured of data confidentiality, anonymity, and the right to withdraw at any time.

## Results

Data analysis led to the extraction of 69 inferential codes, 12 sub-categories, and 3 main categories, including “facing psychological problems”, “attention to emotional reactions”, and “the need to accept and adapt to reality” (Table 4).

**Table 4:** Data analysis results

Sub-category	Main category
Reduced self-esteem	Facing psychological problems
Hatred and self-blame	
Suicidal thoughts and suicide attempts	
Sadness, depression, fear, and panic	Attention to emotional reactions
Despair, anxiety, and confusion	
Anger and hatred towards the opposite sex	
Loss of trust in the opposite sex	
The need to restate the event and express feelings	
Concerns about the inability to get married and ruined future	
The need to return to normal life	The need to accept and adapt to reality
The need to be accepted by parents and spouse	

### Facing psychological problems

Sexual violence is a bitter experience that leads to psychological and emotional instability in women survivors, making it difficult to bear the trauma and leading to severe psychological problems. This highlights the need for support and assistance to build self-confidence while reducing negative emotions in this population. The main category of “facing psychological problems” has the following three sub-categories:

### Reduced self-esteem

Most participants reported negative feelings towards themselves after the rape, illustrating that they did not like themselves anymore and felt worthless. Some survivors believed they had lost their previous abilities and could not deal with many tasks like before.

*“I almost preferred staying home after this incident and did not feel good about myself. I always struggled with myself and couldn’t believe once I gave the senior men and women hope and looked after them in the best way I could.” (45-year survivor)*.

Similarly, service providers believed that women survivors of rape no longer believed in their abilities and needed to be supported to believe in themselves again.

*“Women who experience rape feel broken due to the trauma and think they cannot be present and active in the society as before. I have met survivors who lost their jobs or left school after the trauma as they no longer believed in themselves and their previous abilities. Thus, we must support this population and help them regain their self-esteem and confidence.“ (Gynecologist)*.

### Hatred and self-blame

Hatred and self-blame after the rape were common among the women survivors of rape. Some survivors blamed themselves for illogical trust in others, presence in places and conditions that increased the likelihood of rape (such as parties or business meetings in solitude), and failure to follow safety and self-protection principles. They found themselves guilty of the occurrence of rape.

*“I always tell myself why I trusted. Why? Why? Why? I keep blaming myself for this unwise trust. I was really guilty. I shouldn’t have gone.” (45-year survivor)*.

Service providers identified feelings of self-hatred and guilt as common feelings among rape survivors. They believed that efforts should be made to reduce negative feelings so that they understand they are not the only ones who have experienced this event.

*“These women strongly and constantly blame themselves. They experience a lot of guilt.” (Psychiatrist)*.

*“Many of these women do not feel good about themselves and actually experience self-hatred, which can be due to the pressures imposed by the environment, such as family. We should remind them that they are not the only ones with such an experience and try to alleviate these negative feelings. “(Psychologist)*.

Some survivors changed jobs or separated from their families after experiencing guilt and blame. They mentioned self-blame and guilt as the reasons behind isolation and separation from their family members.

*“I couldn’t continue living with my family anymore. I wasn’t even willing to live in this country, as if everyone blamed me. I really feel guilty. I shouldn’t have gone to that party with my friend. If I hadn’t gone, it wouldn’t have happened.* “(*23-year survivor)*.

The married women survivors of rape preferred to satisfy all their spouses’ demands because they considered themselves guilty.

*“I owe my husband. The assault happened because of my mistake. I am guilty. He (my husband) has the right, and I have to meet all his desires unconditionally.” (30-year survivor)*.

### Suicidal thoughts and suicide attempts

Severe post-rape psychological pressure resulted in suicidal thoughts or behaviors in some survivors. These women found it difficult or unbearable to face blame, insults, and guilt imposed by family and people around them. In addition, dishonor, lack of empathy, and inappropriate behavior of family members frequently led the survivors toward suicidal thoughts to deal with such pressures.

*“… Suicidal thoughts were the first thing that came into my mind. As soon as I got home, I did several self-inflicted wrist cuttings.” (30-year survivor)*.

Some survivors reported suicidal thoughts due to the fear of dishonor since the offender had threatened to release the assault-related films and photos.

*“I had to endure a lot of pressure. I always thought about what to do and how to live with dishonor in this society if (the offender) released the photos and videos, humiliating me in front of my husband and family members. I wanted to commit suicide several times to get rid of these thoughts.” (28-year survivor)*.

Service providers also mentioned the high prevalence of suicidal thoughts or behaviors in women survivors of rape and considered it to be caused by severe psychological problems in them.

*“…Women survivors of rape have repeatedly attempted suicide or committed suicide, which indicates severe and deep psychological problems in them.” (Physician)*.

### Attention to emotional reactions

Emotional reactions of rape survivors can have different forms and should be considered based on their psychological health conditions. The main category of “attention to emotional reactions” has the following six sub-categories:

### Sadness, depression, fear, and panic

Many women survivors of rape suffer from sadness, depression, fear, and panic following rape or even months after it. Sometimes, these problems interfere with various daily affairs, such as sleeping, eating, and social activities. Most survivors regarded their victimization as a source of their sadness and distress.

*“I am very afraid of losing my honor and being judged by others. These people were not involved in my life. What makes me deeply sad is that I have been victimized.” (45-year survivor)*.

Fear in the women survivors of rape was associated with different reasons, such as dishonor in the family and society, loss of job and spouse, etc. Participants narrated the socio-cultural attitudes in stigmas inflicted on women survivors of rape. In this sense, they considered rape as a threat to the loss of their social integrity, reputation, and social status. Some participants reported fear of disgrace as an obstacle to disclosing the assault or seeking services from healthcare centers. There were also instances of the fear of the offender’s threat to continue the relationship sexually.

*“I was afraid of him because I had sent him my photo several times… for example, I had taken some selfies at home and sent them to him. He threatened he would show the pictures to my father or my brother. My father was old, and I was worried he would have a heart attack. “(25-year survivor)*.

Some survivors considered the possibility of being kidnapped, re-injured, or murdered by the same or other offenders as the source of their fear.

*“When I walk outside, every shadow makes me panic and run. I always feel someone, maybe the same offender, wants to injure me.” (27-year survivor)*.

The survivors’ visit to the medical centers was accompanied by the fear of positive STI tests, the results of the virginity and pregnancy tests, etc.

*“I wondered what would happen and what they would say when I was going to lie down on the examination bed (for the virginity test). I could barely conceal my trembling.*” *(25-year survivor)*.

*“I panicked a lot until the results of the tests for the infectious diseases were ready. I wondered what I would do if I had hepatitis or AIDS.” (24-year survivor)*.

### Despair, anxiety, and confusion

Survivors reported negative thoughts and concerns, great confusion, and indescribable daily anxiety after the assault, leaving them almost desperate. They believed post-rape confusion and distress led to their abnormal behavior, aggression, irritability, and lower tolerance threshold.

*“I was in a bad mood and didn’t have normal conditions. I felt confused and miserable. I behaved aggressively to my children and hit them twice, sitting and crying after each time.” (29-year survivor)*.

Also, service providers found socio-cultural components to be effective in reducing the social value and dishonor of women survivors of rape as well as their anxiety and despair.

*“These people are very anxious on the first days of treatment since it is difficult for them to accept such an experience, making them desperate.” (Psychiatrist)*.

Two survivors who had got pregnant and aborted the fetus following rape reported anxiety and confusion about the fate of their pregnancy. The dishonor and disgrace resulting from pregnancy made them more anxious, which was the main reason behind abortion.

*“When my pregnancy test was positive, the only thing that came into my mind was to get rid of the child who could be a source of dishonor. How could I live with such disgrace and shame? I didn’t know where to go or whom to speak about my pregnancy. I felt miserable and stressed.” (27-year survivor)*.

### Anger and hatred towards the opposite sex

The survivors had anger and hatred towards other men due to the rape and victimization by a man. As stated by the survivors, this feeling made communication with the spouse, brother, or father irritating and challenging for them.

*“When I go to a store whose salesperson is a man, I have no control over my hatred and cannot behave normally. I don’t know why men make me feel sick. I hate all men and think they are all the same.*” *(45-year survivor)*.

Some survivors also believed their anger and hatred stemmed from their inability to defend themselves and their lack of right to prove the crime of assault.

*“I had a deep desire for revenge and wanted to ruin his life. I couldn’t get my right as I deserved it, so why not relieve my pain through revenge? I could even call his father and threaten him.*” *(30-year survivor)*.

### Loss of trust in the opposite sex

As stated by the survivors, they could no longer trust men after the assault. The loss of trust in the opposite sex was especially reported by women who had regarded the offender as reliable and visited him in a work meeting, party, café, etc., leading to their victimization.

*“I can’t do my shopping over the phone as I did before. I used to order from several shopkeepers, but I am afraid they may misbehave when delivering my orders. I cannot trust them anymore.” (23-year survivor)*.

Also, service providers narrated that women survivors of rape usually have a sense of distrust towards men due to the trauma they have suffered, and therefore rebuilding trust in them should be considered.

*“…One of the actions that should be taken into consideration in the counseling sessions for these women is rebuilding trust towards the male sex.” (Psychologist)*.

### The need to restate the event and express feelings

Most survivors considered their need to disclose the incident, be heard, express their feelings, and release emotions the most critical factor. They also emphasized their need for a reliable, empathetic person who supported them without judgment. For some survivors, this person was their friend or a counselor.

*“I needed someone to whom I could talk and in whose arms I could cry; someone who could help me and tell me what to do in such miserable conditions; someone who could calm me down; someone who wouldn’t consider me guilty because I shouldn’t have gone out at that time of night.*” *(23-year survivor)*.

Similarly, service providers emphasized the need for women survivors of rape to disclose the incident to a trusted person.

*“One of the basic needs of these women is to empty themselves, trust someone, and disclose what has happened to them.*” *(Psychiatrist)*.

### Concerns about the inability of getting married and ruined future

Avoiding extramarital sexual relationships and maintaining virginity before marriage are essential cultural requirements for women to get married in Iran. Thus, many single women survivors of rape, who had lost their virginity, were unwilling to get married or had doubts about it due to fear of facing numerous challenges, such as the stigma of losing their virginity and moral deviations. They were worried about their future, considered it ruined, and saw themselves unable to get married.

*“I don’t want to get married anymore. I don’t want to face the challenge of my loss of virginity. I have no future. My future is ruined*.” *(23-year survivor)*.

*“Certainly, if someone finds out what has happened to me before marriage, he will break up. Even if I get married, and my husband discovers I am not a virgin, he will think I am corrupted. Thus, I wonder whether I should get married or not.*” *(29-year survivor)*.

Similarly, service providers narrated that in the Iranian culture, a girl’s virginity for marriage is still vital for families, and they attributed the reluctance to get married among rape survivors to this reason.

*“Many of these women do not get married because they are no longer virgins. They are worried about their honor due to virginity loss and decide to remain single to face fewer challenges in the future.*” *(Reproductive health specialist)*.

### The need to accept and adapt to reality

Rape survivors should accept the event and return to their former life. Acceptance of the parents and spouse, along with support and empathy, is also a fundamental need. The main category of “the need to accept and adapt to reality” includes two sub-categories:

### The need to return to normal life

The survivors wanted to return to their pre-assault peaceful life after experiencing much stress and tension related to the rape. They strove to find effective solutions, such as psychological support from family and friends, to deal with bitter memories, harm, and discouraging thoughts.

*“I wish someone could help me to get rid of these thoughts and nightmares. I yearn for an untroubled night’s sleep without fear or worry. I miss my previous peaceful life.*” *(44-year survivor)*.

Service providers also emphasized the need for women survivors of rape to achieve lost peace.

*“… Many of these women refer to us after a while and want to visit a psychiatrist. They constantly complain about their unfavorable conditions and strive for their lost peace.*” *(Social worker)*.

The need to receive online and face-to-face counseling from psychologists, social workers, and psychiatrists, drug therapy, and spiritual and religious support were among the main requirements and solutions raised by the survivors to return to their normal lives.

*“… I start praying and reciting Quran to feel better at times when my disappointing thoughts rush over me and make me feel miserable. Sometimes I go to the shrine and sit there for hours.*” *(39-year survivor)*.

### The need to be accepted by parents and spouses

According to the service providers, the correct reaction of parents, spouses, and others contributes significantly to accepting and not rejecting the survivor. Some survivors expressed their concerns about and dissatisfaction with the blame by their spouses and parents and their negative looks. The survivors needed confirmation, acceptance, empathy, and support without judgment.

*“Most survivors refer to us alone. I think parents and family members, especially brothers and fathers, cannot tolerate the presence of their daughter or sister after the assault and fail to support her in our society. These girls are usually under significant pressure and tension from their fathers and brothers.*” *(Reproductive health specialist)*.

Although some survivors were frequently blamed by their parents or spouses after disclosing the event, they were distressed due to the sufferings of their family members resulting from the assault-related dishonor. Thus, many women survivors of rape tried to spend less time at home and among family members.

*“Each time my father looked at me, he murmured: You are the source of our shame and dishonor. After all these years, I wish I had never seen such a day.” (21-year survivor)*.

In this regard, most survivors reported unsatisfactory relationships and behaviors of their parents or spouses after the rape. In many cases, the lack of sympathetic relations with parents or spouses hindered disclosing the incident and asking for support.

*“I’d like him to support me to deal with the bitterness and pain of this event. On the other hand, I knew he would become distressed and could not tolerate such pressure. He might have even divorced me, so I preferred not to say anything.*” *(28-year survivor)*.

## Discussion

The present study was done to highlight the psychological needs of women survivors of rape. The results revealed that facing psychological problems, attention to emotional reactions, and the need to accept and adapt to reality were the main psychological needs of this population.

Based on the results, women survivors of rape had to face various psychological problems, such as reduced self-esteem, hatred and self-blame, suicidal thoughts, and suicide attempts. According to Krahé et al., rape is a risk factor for depression as a general indicator of psychological health and reduced self-esteem as a specific outcome [22]. Rape survivors suffer from diminished self-esteem, which can be a risk factor for re-victimization, necessitating serious interventions to strengthen their abilities and improve self-esteem [23]. It seems that tensions imposed by the family and others play a critical role in self-blame, hatred, and reduced self-esteem in the survivors. Thus, women survivors of rape need individual and environmental interventions immediately after the assault to enhance their self-confidence in managing self-blame and hatred while also improving their self-esteem.

According to the results, some survivors reported suicidal thoughts and unsuccessful suicide attempts to deal with the psychological and social pressures imposed by their parents, spouse, and society. Abrahams et al. observed that many women survivors of rape had suicidal thoughts, and 1 in 8 continued to have suicidal thoughts during the next two years, revealing survivors’ ongoing psychological post-assault distress [24]. Maisha et al. showed that rape survivors were blamed for issues that might have influenced the offender’s behavior, such as high-risk socialization, alcohol consumption among several boys, wearing provocative clothes, late reports of the assault, sending different messages to boys in virtual media, etc. [25]. Rape stereotypes are social messages that lead women to perform predetermined gender roles regarding sexual behaviors and lead to the formation of rape culture [26]. It seems that removing these stereotypes from the cultural components of society can result in the liberation of society from the culture of rape. Also, rape survivors need the special attention of psychotherapists to identify and manage their suicidal thoughts and attempted suicide.

The present study indicated that women survivors of rape needed attention to different emotional reactions. Many survivors were depressed and distressed even months after the incident. Nöthling et al. reported a 48.5% incidence of PTSD in the first three months after rape and a 54.8% cumulative incidence in six months following the incident. This study reported the stigma of rape (guilt, shame, self-blame, reduced social value, and dishonor) and depression over time as the main predictors of PTSD [27]. Thus, close attention to the symptoms of grief, depression, and distress seems necessary immediately after the assault, indicating the importance of careful supervision and proper treatment to reduce the severity of PTSD and prevent long-term consequences.

As found by the present study, rape subjects the survivors to many fears. Women survivors of rape have to deal with different fears surrounding them, disrupting their daily activities. Several studies have shown that social fear of dishonor and disgrace stems from socio-cultural components [25, 28]. Widanaralalage et al. indicated that due to stigma and hostility, rape victims do not report the incident to the police and are denied access to medical care [10]. Schmitt et al. showed that 33% of women survivors in the Congo considered rape a threat to their social integrity, reputation, and social status, confirming the role of socio-cultural attitudes in stigmas inflicted on women survivors [29]. The General Strain Theory (GST) was proposed by Agnew (1992) concerning the fear of re-victimization by the offender, revealing that survivors are prone to re-victimization due to the experience of trauma and intense psychological pressure [30]. Constantin and Boyett also emphasized this issue and showed that the survivors experienced pressures such as shock, fear, restlessness, and lack of emotional support, leading to their ongoing concerns about re-victimization under severe psychological pressure [31]. Archer et al. examined fears experienced by women survivors of rape and reported an increase in their post-assault protective behaviors to prevent re-victimization [32]. According to Maisha et al., the survivors were emotionally distressed and lived with the fear of re-victimization, constantly preventing their presence in society [25]. Thus, survivors need interventions to increase their comfort and reduce the psychological pressure to control their fear.

As revealed by the present study, the survivors suffered from negative thoughts, numerous concerns, and distress, making them anxious and desperate. Carey et al., in a study on 483 female first-year students, found sexual assault during the first semester predicted clinically significant levels of anxiety and depression at the end of that semester [33]. The present study found anger, hatred, and loss of trust in the opposite sex as the most common feelings experienced by women survivors of rape and required to be addressed. Similarly, Shepp et al. showed that the survivors experienced adverse feelings and emotions towards the offender, including post-rape anger and revenge [34]. Rothman showed less serious emotional relationships between girls with a history of rape in university and the opposite sex in the coming years. The women survivors of rape also reported less emotional and sexual intimacy nine years after the incident [35]. Thus, women survivors of rape face numerous problems in establishing and maintaining relationships with men due to their hatred and anger towards the male sex, highlighting the importance of comprehensive interventions.

Another important and primary need of women survivors of rape in the present study was recounting the incident and expressing their feelings. Buchbinder et al. showed that many women survivors of rape needed to be heard and express their feelings to others [36]. The reactions received by the survivors after the assault disclosure are significant. Failure to support while blaming the survivor for the incident can lead to numerous adverse outcomes. Blaming and negative social responses are disturbing for women survivors of rape [37, 38] and are associated with increased self-blame, reduced sexual assertiveness, social isolation, and depression [37]. Recent studies have shown a significant relationship between depressive symptoms in survivors and blameful reactions to disclosure of rape [37, 39]. It seems that this catharsis abreaction is critical in maintaining confidentiality and providing the conditions for communication with experienced experts and consultants since the involvement of people with insufficient expertise can lead to numerous adverse consequences, such as wrong guidance, blame, and stigma. Thus, interventions are required to encourage the rape survivors to recount the incident to the experts and experienced professionals as quickly as possible and receive supportive, empathetic, and efficient services.

The present study also highlighted the contribution of rape to severe anxiety in girls regarding marriage in the future as it leads to loss of virginity (which is greatly important in the Iranian culture). In many cultures, virginity has been a symbol of pride, dignity, and honor, motivating girls to get married,which is promoted through different national, cultural, and religious programs [40]. Although WHO has emphasized eliminating the “virginity test” before marriage, it is still prevalent in many countries, including Iran. In addition, even in areas where this test is not performed, the virginity of girls and avoiding sex before marriage is still of considerable importance. Many studies have emphasized that “virginity tests” are worthless and violate women’s rights [40, 41]. As part of the global call to eliminate violence against women and girls everywhere, on 17 October 2018, The UN Human Rights Office, UN Women, and WHO issued a statement stressing that ‘virginity testing” is unscientific and a violation of human rights and that this medically unnecessary, and often painful, humiliating and traumatic practice must end [42]. Considering the deep connection between virginity and culture in some societies, including Iran, cultural and social interventions can contribute to addressing the concerns and despair of women survivors of rape concerning their future marital status.

The present study indicated that women survivors of rape needed to accept reality, including their return to normal life and acceptance by parents and spouses. On the other hand, although women survivors of rape need acceptance by their parents and spouses as well as their comprehensive support to accept the reality of the incident, parents and spouses usually fail to provide such conditions. Ullman et al. believed survivors did not disclose the incident due to the fear of adverse social reactions, lack of support, family expectations, social norms, and problematic gender responses [38]. It seems that these women required empathetic acceptance, positive family and spouse relations, and comprehensive support services to control internal and environmental stressors, establish relative peace, and return to their previous life. This goal can be realized through educational interventions to empower the survivors’ families.

### Implications and recommendations

Recognizing the psychological needs of women survivors of rape can facilitate evidence-based policy-making and provide the basis for targeted training, appropriate programs and guidelines, and support for these individuals. The results of this study can be presented to the health policymakers of the country to make necessary changes in the health system to improve the psychological health of women survivors of rape. In addition, the results of this study can be of great use in understanding, evaluating, and recognizing the psychological needs of women survivors of rape by sensitizing health service providers (midwives, gynecologists, psychiatrists, and psychologists) and drawing their attention to these needs. In this context, health service providers can identify the psychological needs of women survivors of rape and try to fulfill these needs and improve the psychological health of these women. Meanwhile, policy-making in education through social media and other organizations to change the cultural components of society can lead to increased social support for women survivors of rape. The results of this study are expected to be used as a basis for future research in this field and will lead to the identification of newer research areas. In this regard, one of the research areas is designing and developing programs to improve psychological health in women survivors of rape, which can be done based on the research results.

### Limitations of this study

Considering the cultural taboos surrounding rape in Iran and the sensitivity of the subject under study, the present study was limited by the problems the survivors faced in recounting the incident due to their shame and embarrassment. Thus, attempts were made to explain the importance of the research, provide the participants with a safe and calm environment, establish effective communication, and subsequently gain their trust. However, this case can be one of the limitations of the present study. This study was done in Isfahan city. Although the socio-cultural situation of this city is similar to that of many regions of Iran, the results of this qualitative study cannot be generalized to the entire country. However, efforts were made to enhance the rigor and trustworthiness of the findings by applying multiple and different methods.

## Conclusion

According to the results, the main psychological needs of women survivors of rape included facing psychological problems, attention to emotional reactions, and the need to accept and adapt to reality. Therefore, to fulfill the psychological needs of these women, supportive and psychological interventions can be considered in both individual and environmental dimensions. In the individual dimension, interventions can be done to improve the survivors’ psychological state. Interventions in the environmental dimension can be implemented to reduce family and social stressors. Also, eliminating gender stereotypes affecting the occurrence of rape in the components of Iranian culture can lead to the liberation of the society from the culture of rape.

## Data Availability

The datasets generated and/or analyzed during the current research are not publicly available as individual privacy could be compromised but are available from the corresponding author on reasonable request.

## Abbreviations

WHO: World Health Organization
PTSD: Post-Traumatic Stress Disorder
DIC: Drop-in Centers
GST: General Strain Theory
